# Liver fatty acid-binding protein might be a predictive marker of clinical response to systemic treatment in psoriasis

**DOI:** 10.1007/s00403-019-01917-w

**Published:** 2019-04-16

**Authors:** Anna Baran, Paulina Kiluk, Magdalena Maciaszek, Magdalena Świderska, Iwona Flisiak

**Affiliations:** 1 Department of Dermatology and Venereology, Medical University of Bialystok, Zurawia 14 St, 15-540 Białystok, Poland; 2 Department of Infectious Diseases and Hepatology, Medical University of Bialystok, Zurawia 14 St, 15-540 Białystok, Poland; 3 Department of Physiology, Medical University of Bialystok, Mickiewicza 2C St, 15-222 Białystok, Poland

**Keywords:** Psoriasis, Fatty acid-binding proteins, Lipids, Adiposity, Liver

## Abstract

Fatty acid-binding proteins play an inconclusive role in lipid metabolism and cardiometabolic diseases (CMDs) which are closely related with psoriasis. Aim of the study was to investigate the diagnostic value of serum liver fatty acid-binding protein (FABP1) level and associations with disease severity, inflammation or metabolic parameters and influence of systemic treatment in psoriatic patients. The study included thirty-three patients with active plaque-type psoriasis and eleven healthy volunteers. Blood samples were obtained before and after 12 weeks of therapy with methotrexate and acitretin. Serum FABP1 concentrations were analyzed by the enzyme-linked immunosorbent assay. Statistical analysis was performed for correlation of FABP1 with anthropometric, metabolic or inflammatory indices and treatment used. Serum liver-type FABP levels were significantly increased in psoriatic patients compared to the controls (*p* < 0.001). No statistical correlations between FABP1 and PASI (*p* = 0.25) was noted, however patients with severe psoriasis had the highest level of FABP1. No significance with metabolic parameters was obtained, beside a positive significant relation with BMI after therapy (*p* = 0.03). Liver-type FABP significantly correlated with CRP (*p* = 0.01) and morphotic blood elements. Systemic treatment combined resulted in significant decrease of FABP1 (*p* = 0.04), regardless of the drug: *p* = 0.1 in acitretin group, *p* = 0.3 in methotrexate group. Liver-type FABP might be a novel marker of psoriasis and predictor of clinical response to systemic therapy. FABP1 could be involved in CMDs risk assessment and perhaps link psoriasis with hematological disorders.

## Introduction

Psoriasis is a systemic chronic inflammatory disease affecting 2–4% of the world population. It involves primarly the skin, however significant associations with numerous comorbidities including obesity, cardiometabolic diseases (CMDs), metabolic syndrome (MS), diabetes mellitus (DM) or non-alcoholic fatty liver disease (NAFLD) have been demonstrated [11, 12]. A number of studies have shown higher rate of hypertension, MS or DM within the severity of psoriasis [1, 11, 12, 17]. The mortality rate of psoriatics is increased compared with the general population due to increased risk of cardiovascular disorders, mainly myocardial infarction (MI) and thromboembolic events [1, 17, 18]. The relationship of psoriasis and its comorbidities is multifactorial and still to be elucidated. Common mechanisms which have been shown are chronic systemic inflammation, genetic basis, immune pathways, insulin resistance (IR), atherosclerosis, angiogenesis, oxidative stress, secretion of adipokines and other bioactive molecules or dyslipidemia [6, 17, 18]. Lipid homeostasis disturbances take part in the inflammatory or metabolic signaling pathways and contribute to the development of CMDs, including psoriasis [4, 5, 14, 27].

Fatty acid-binding proteins (FABPs) are a family of 14–15 kDa proteins involved in the regulation of lipid trafficking and inflammation particularly through protection against the harmful accumulation of long-chain fatty acids (FA) [5, 14]. To date at least nine isoforms have been identified and named according to the tissues active in lipid metabolism: liver (L-), intestinal (I-), heart (H-), adipocyte (A-), epidermal (E-), ileal (Il-), brain (B-), myelin (M-) and testis (T-). FABPs have been recognized as promising indicators of different organs damage but also related with development of immunometabolic diseases [10, 14].

So far, in psoriasis the role of only one FABPs isoform—epidermal fatty acid binding protein (E-FABP, FABP5) has been more widely documented. Studies demonstrated its impact on the development of MS, regulation of insulin sensitivity, lipid homeostasis or differentiation of keratinocytes and its overexpression in psoriatic plaques or atopic dermatitis [10, 14].

From the remaining FABPs only heart—(H-FABP, FABP3) and adipocyte fatty acid-binding protein (A-FABP, FABP4) were evaluated in patients with psoriasis in the research conducted by us [5]. Heart-type FABP is an extremely sensitive marker of MI and a predictor of coronary heart disease in MS patients and is elevated in DM [10, 14]. We did not confirm the possible involvement of FABP3 in the risk assessment of CMDs in patients with psoriasis, but we have indicated possible link with chronic inflammation and liver dysfunction [5].

Adipocyte fatty acid-binding protein has been shown to be elevated in patients with obesity, DM, MS, NAFLD and cardiovascular diseases (CVD) [5, 14, 22, 32]. FABP4 has been recently linked with increased cardiovascular morbidity and mortality, which is also observed in psoriasis [32]. Increased FABP4 level is an important cardiometabolic predictor, prognostic factor in end-stage renal disease or coronary artery disease (CAD) [9, 16, 32, 35]. In our previous research serum FABP4 level was significantly increased comparing to healthy controls. We concluded that adipocyte-type FABP could be one of the markers of psoriasis, however further studies are needed to elucidate its possible role in linking with immunometabolic diseases [5].

Liver-type fatty acid binding protein (L-FABP, FABP1) the first isoform discovered, highly expressed in the liver, but also in the lung, pancreas, intestine and kidney, accounts for 5–11% of cytosolic proteins [21]. Beside its pivotal role in FA uptake and utilization, FABP1 has multiple ligand binding properties and thus cytoprotectant role [38]. It is an early biomarker of acute kidney injury or chronic kidney disease, liver and lung damage or a promising indicator for detecting NAFLD which co-occurs in up to 50% of psoriatics [3, 21, 24, 38]. Moreover, chronic inflammation present in psoriasis leads to hepatic steatosis and further comorbidities as MS or cardiovascular events more frequent in both diseases [3, 24]. Recent literature has pointed that genetic variations of FABP1 modulate the risk of NAFLD and the isoform reduction in the liver of the mice was associated with decreased inflammatory and oxidative stress markers [21, 26, 30, 38]. These promising outcomes suggest a possible role of FABP1 in the liver-skin axis, and presumably in psoriasis, the more it might protect from oxidative stress in a multifarious manner over-present in that dermatosis and modulates the action of PPARs (peroxisome proliferator-activated receptors) which are expressed in psoriasis as well [4, 38]. Liver-type FABP was suggested to be independently related with aortic stiffness in patients with CAD compared to healthy controls what additionally may link psoriasis with CMDs [29].

To our knowledge, serum liver-type FABP has not been investigated in psoriasis so far. Therefore, we aimed to assess serum FABP1 levels in patients with active plaque-type psoriasis and its relationship with the disease severity, inflammatory or metabolic markers and variations after systemic therapy. Further, the objective of the study was the determination of diagnostic value of FABP1 and potential use in prediction of liver damage or the risk of cardiometabolic diseases in patients with psoriasis.

## Materials and methods

The prospective study was conducted on thirty-three patients (21 males and 12 females) with active plaque-type psoriasis, at median age 58 (44–63 years) and 11 sex-, age- and BMI-matched healthy volunteers. The study was approved by the local Bioethical Committee and was in accordance with the principle of the Helsinki Declaration. Written informed consents were obtained from every participant before the initiation. None of the patients or controls were under any dietary restriction. The patients did not use any systemic treatment for at least 3 month prior to enrollment. The exclusion criteria included other types of psoriasis, inflammatory conditions, autoimmune or cardiometabolic diseases and malignancy. Psoriasis area and severity index (PASI) was assessed by the same investigator in all subjects. The study group was divided according to severity of psoriasis into three sub-groups: mild (PASI 1) < 10 points, moderate (PASI 2) between 10 and 20 and severe (PASI 3) > 20. Body mass index (BMI) was calculated as weight/height^2^ (kg/m^2^). All participants were also divided into sub-groups depending on BMI, group 0 meant the healthy controls, BMI 1 was related to normal-weight (BMI 18.5–24.9) and consisted of 10 persons, group 2—overweight (BMI 25–29.9) present in 9 psoriatics, BMI 3—obesity (BMI > 30) noted in 14 patients. Laboratory tests including C-reactive protein (CRP), complete blood count (CBC), serum glucose, total cholesterol (Chol), HDL, LDL and triglycerides (TG), transaminases were performed prior to and after treatment. The patients were started two systemic medications: 19 persons with methotrexate (MTX) 15 mg/week using folic acid supplementation (15 mg/week, 24 h after MTX intake) and 14 subjects with acitretin at a dose 0.5 mg/kg/day. The treatment period lasted 12 weeks.

### Serum collection

Fasting blood samples were obtained from healthy subjects and patients before and after 12 weeks of treatment using vacutainer tubes with clot activator. Samples were centrifugated at 2000 × *g* for 10 min and stored at − 80 °C until analyses. Liver fatty acid-binding protein levels were measured using enzyme-immunoassay kit supplied by Quantikine^®^ R&D Systems, Minneapolis, USA. The limit of detection for FABP1 was 0.58 ng/ml and the standard curve ranges was 1.56–100 ng/ml. Optical density was read at a wavelength of 450 nm. The concentrations were assessed by interpolation from calibration curves prepared with standard samples provided by the manufacturer.

### Statistical analysis

Data analysis was performed using the GraphPad Prism5 (La Jolla, CA) and Statistica 12.0 software, a value of *p* < 0.05 was regarded statistically significant. Data are statistically expressed as the mean ± standard deviation (± SD), median value, confidence interval (25–75% percentile, CI) and percentage when appropriate. Comparisons between the groups were performed using Student’s independent paired *t* test. Correlations were performed using Mann–Whitney Rank Sum test and the Spearman coefficient correlation for not normally distributed variables. For quantitative data divided in categories, one-way ANOVA Kruskal–Wallis test was used. Logistic regression was used for multivariate analysis of associations.

## Results

Clinical, demographic and laboratory characteristics regarding the study group are summarized in Table 1. A total of 33 patients with active plaque-type psoriasis, 22 men and 11 women with the mean age of 54 (24–85 years) and 11 age-, sex- and BMI-matched healthy volunteers were enrolled to the study. Median value of BMI was 26.9 kg/m^2^ (23.9–33.6). Median of basal PASI score was 15 (11–24.1) points and 3.4 (2.4–6.4) after treatment. In the study group 6 patients (18.2%) had mild psoriasis (PASI < 10), 14 (42.4%)—moderate (PASI 10–20) and 13 (39.4%) were diagnosed with severe form (PASI > 20). Seventeen subjects (51.5%) reported positive family history of psoriasis, more often males (12 vs. 5 women), 15 psoriatics (45.4%) were current cigarette smokers, 12 men and 3 women.

**Table 1:** Selected characteristics of the patients

Characteristics	Median	25% percentile	75% percentile	Mean	SD
Sex (male/female)	21/12	–	–	–	–
Age (years)	58	44	63	54	16.8
PASI before treatment	15	11	24.1	17.2	7.8
PASI after treatment	3.4	2.4	6.4	4.6	3.2
BMI (kg/m^2^)	26.9	23.9	33.6	28.9	6.4
WBC (× 10^3^/ml)	7	6.3	8.3	7.5	1.9
PLT (× 10^3^/ml)	244	202.5	304	250.7	73.2
Glucose level (mg/dl)	84	78	100.5	93.3	29.8
CRP (mg/l) before treatment	5.1	1.9	9.7	8.6	9.5
CRP (mg/l) after treatment	1.9	1.1	3.7	3.2	3.4
ALT (U/l)	24	14	30.5	24	11.8
AST (U/l)	19	13.5	26	20.9	9.3

**Fig. 1:**
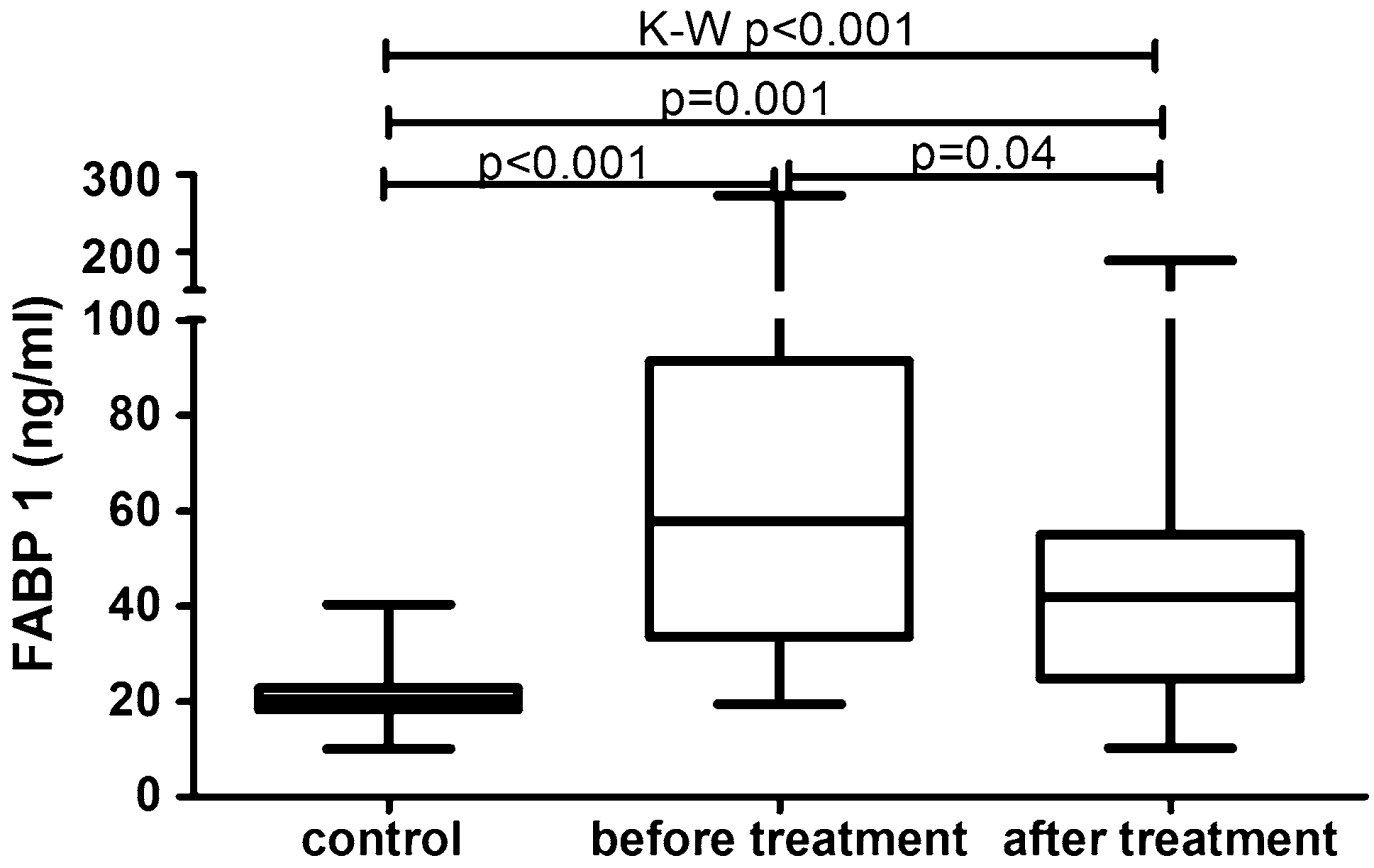
Comparison between FABP1 concentrations before and after total treatment in psoriatics patients and in controls. Value of *p* < 0.05 was considered statistically significant after Student *t*-test or Kruskal–Wallis analysis

**Fig. 2:**
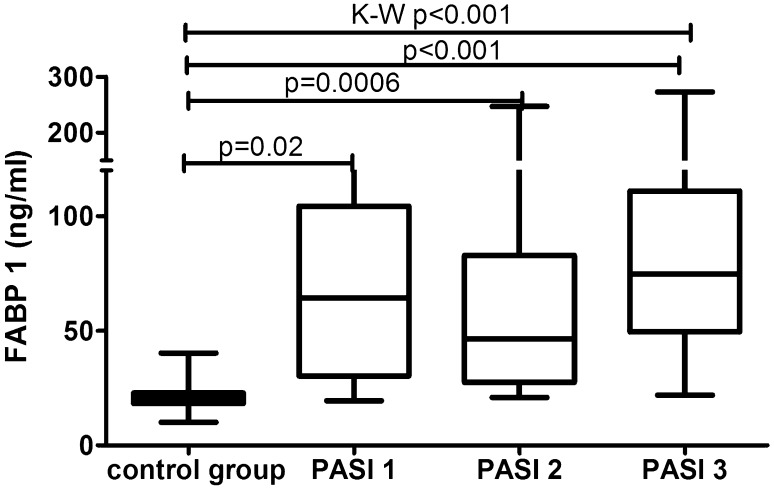
Comparison between serum FABP1 concentrations depending on PASI before treatment and controls. Value *p* < 0.05 was considered statistically significant with use of Kruskal–Wallis analysis and Mann Withney test

**Fig. 3:**
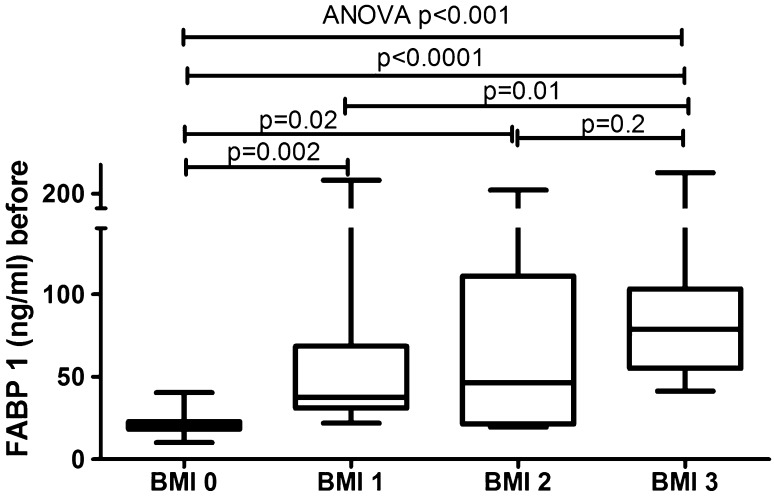
Comparison of serum FABP1 concentrations between sub-groups depending on BMI before treatment. Value *p* < 0.05 was considered statistically significant

The median of liver fatty acid-binding protein level was 57.8 (33.6–91.4) ng/ml and was strongly significantly higher (*p* < 0.001) than of the controls—20.5 (18.3–22.9) ng/ml (Fig. 1). FABP1 levels regarding psoriasis activity were significantly increased in all three sub-groups comparing to the controls: PASI < 10 (*p* = 0.02), PASI 10–20 (*p* = 0.0006) and PASI > 20 (*p* = 0.001) (Table 2, Fig. 2). There was no significance between those three sub-groups (Table 3) Serum liver-type FABP concentration did not correlate with psoriasis severity expressed through PASI score (*p* = 0.25) (Table 4). No significance in terms of gender was noted. Comparisons between FABP1 levels in females versus males with use of Mann–Whitney test were as follows: *p* = 0.2 before treatment and *p* = 0.7 after. Liver-type FABP correlated marginally positively with age of the study group before (*p* = 0.047) and after therapy (*p* = 0.014) (Table 4). Further, negative significant correlations of FABP1 with RBC, hemoglobin (HGB) and hematocrit (HCT) levels were noted, both before and after therapy (Table 4). Serum liver fatty acid-binding protein correlated positively with C-reactive protein at statistical significance before treatment, losing the one after (*p* = 0.01, *p* = 0.37, respectively) (Table 4). Regarding liver enzymes, a significant positive correlation between FABP1 and ALT (alanine aminotransferase) after treatment was shown (*p* = 0.02) (Table 4). Of metabolic indices such as glucose level, lipids profile or BMI no statistical importance was noted, beside a significant positive correlation between FABP1 and BMI after the treatment (*p* = 0.03) (Table 4). Considering the division of the study population depending on BMI, liver-type FABP was significantly elevated in all psoriatic sub-groups compared to controls (Fig. 3). The significance was the highest in obese patients as compared to the controls (*p* < 0.0001, ANOVA *p* < 0.001) (Fig. 3).

**Table 2:** Comparison of mean (SD) of concentrations of serum FABP1 in controls versus psoriatic patients depending on PASI score before treatment

	Controls	Psoriatic patients		
		< 10	10–20	> 20
	*n* = 10	*n* = 6	*n* = 14	*n* = 13
	Mean (SD)	Mean (SD)	Mean (SD)	Mean (SD)
FABP1 (ng/ml)	20 (18–22)	64 (30–104)	46 (27–82)	75 (49–111)
*p*		0.02	0.0006	0.0001

Value of *p* < 0.05 was considered statistically significant using Student *t*-test and Mann–Whitney test

**Table 3:** Serum FABP1 concentrations before and after treatment in psoriatic patients between three sub-groups depending on PASI

	PASI		
	< 10 versus 10–20	< 10 versus > 20	10–20 versus > 20
*Before treatment*			
FABP-1	0.70	0.50	0.07
*After treatment*			
FABP-1	1.0	0.76	0.63

Value of *p* < 0.05 was meant statistically significant using the *t*-Student test and the Mann–Whitney test

**Table 4:** Main variables of the study in patients before and after treatment and correlations with serum FABP1 levels

Characteristics	FABP1 before treatment	FABP1 after treatment
	*R*, (*p* value)	*R*, (*p* value)
Disease duration	0.15, (0.41)	− 1.43,(0.42)
Age (years)	0.33, (0.047)*	0.43, (0.014)*
Height	− 0.20, (0.25)	− 0.19, (0.27)
Weight	0.15, (0.39)	0.26, (0.14)
BMI	0.31, (0.07)	0.37, (0.03)*
PASI	0.20, (0.25)	− 0.16, (0.36)
CRP (mg/l)	0.43, (0.01)*	0.16, (0.37)
WBC (× 10^3^/ml)	− 0.06, (0,72)	− 0.23, (0,20)
RBC (× 10^3^/ml)	− 0.44, (0.01)*	− 0.37, (0.03)*
HGB	− 0.49, (0.004)*	− 0.46, (0.007)*
HCT	− 0.38, (0.03)*	− 0.36, (0.04)*
PLT (× 10^3^/ml)	0.11, (0.53)	− 0.07, (0.70)
ALT (IU/l)	0.18, (0.30)	0.40, (0.02)*
AST (IU/l)	− 0.05, (0.77)	0.05, (0.77)
Glucose (mg/dl)	0.12, (0.52)	− 0.27, (0,13)
Cholesterol (mg/dl)	− 0.23, (0.19)	− 0.12, (0.48)
HDL-C (mg/dl)	0.14, (0.68)	0.07, (0.94)
LDL-C (mg/dl)	− 0.18, (0.30)	− 0.05, (0.82)
TG (mg/dl)	− 0.11, (0.54)	− 0.10, (0.59)

*Statistically significant correlation (*p* < 0.05) versus controls (Mann–Whitney test)

**Fig. 4:**
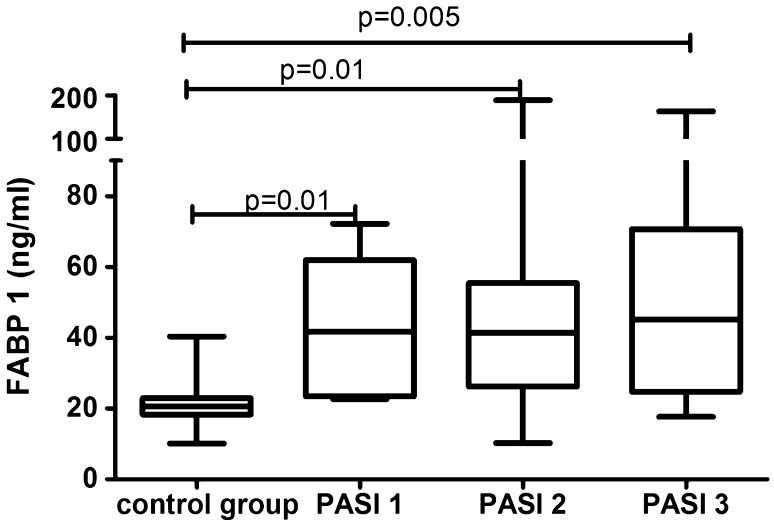
Comparison between serum FABP1 concentrations depending on PASI after treatment and controls. Value *p* < 0.05 was considered statistically significant with use of Mann–Whitney test

**Fig. 5:**
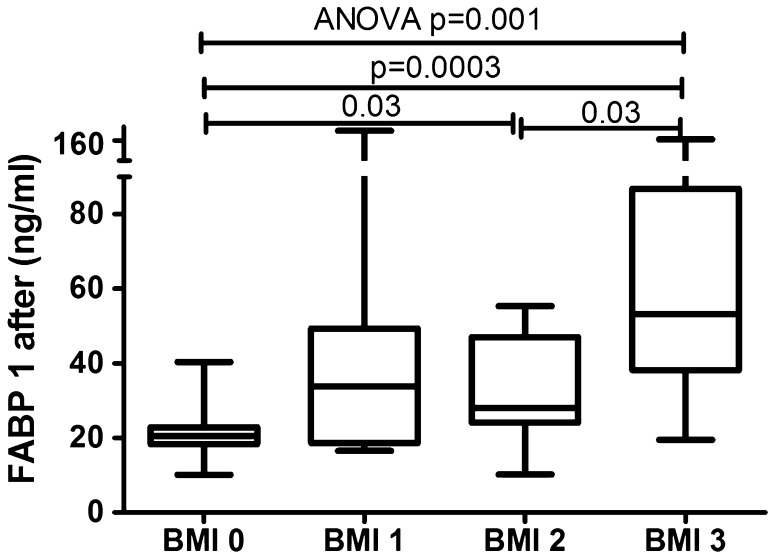
Comparison of serum FABP1 concentrations between sub-groups depending on BMI after treatment. Value *p* < 0.05 was considered statistically significant with use of Mann–Whitney test and ANOVA test

After 12 weeks of systemic therapy the skin lesions in all studied patients have improved. The median of total PASI score decreased from basal PASI 15 (11–24.1) to 3.4 (2.4–6.4) after total therapy (Table 1). The median FABP1 significantly decreased (*p* = 0.04), remaining statistically higher than of the controls (*p* = 0.001) after two drugs in total and the level was 41.9 (24.8–55.1) ng/ml (Fig. 1, Table 5). After separation into subgroups of patients treated with both drugs separately, liver-type FABP concentration decreased after acitretin over twice, however without statistical significance (*p* = 0.1) as well as after methotrexate (*p* = 0.3) where the level remained almost the same as before (Table 5). With regard to the severity of psoriasis, serum liver-type FABP was still statistically elevated in all three PASI subgroups versus controls, respectively: PASI 1 (*p* = 0.01), PASI 2 (*p* = 0.01), PASI 3 (*p* = 0.005) (Fig. 4). There were no important differences in FABP1 levels between three PASI sub-groups after treatment (Table 3). According to BMI, FABP1 level remained significantly higher in all three sub-groups comparing to the controls and the significance was still the highest in obese patients (*p* = 0.0003) (Fig. 5).

**Table 5:** The median values and confidence intervals (CI) of serum FABP1 concentrations in psoriatic patients before and after total treatment and after acitretin and methotrexate separately

		Total treatment	Acitretin	Methotrexate
		Median (CI)	Median (CI)	Median (CI)
FABP1 (ng/ml)	Before treatment	57.8 (33.6–91.4)	62.7 (21.9–99.1)	56.7 (41.4–86.1)
	After treatment	41.9 (24.8–55.1)	27.1 (18.6–44.4)	49.2 (36.9–86.1)
	*p* (before vs. after treatment)	0.04	0.1	0.3

Statistical analysis with Mann–Whitney test, *p* < 0.05 statistically significant

## Discussion

To current literature, this is the first prospective study evaluating serum liver fatty acid-binding protein levels in patients with psoriasis and what adds value, additionally with regard to systemic treatment. We were also the first who evaluated adipocyte and heart acid-binding protein in psoriatics, however in relation with topical therapy [5]. Previously we have highlighted that adipocyte-type FABP might be a biomarker of psoriasis and heart-type-FABP could be associated with chronic inflammation or liver disorders in psoriatics. These FABPs presumably are not useful in assessing psoriasis severity and the effectiveness of antipsoriatic topical treatment [5]. More research papers have proved importance of epidermal-type FABP in the dermatosis, also during different methods of treatment, however the results were still single, inconsistent and incomparable [23, 25].

A number of studies investigated liver fatty acid-binding protein suggesting its role in systemic metabolism, obesity and other cardiometabolic diseases [21, 34, 38]. In this study, we demonstrate for the first time that serum liver fatty acid-binding protein level is markedly elevated in patients with psoriasis compared to healthy controls. Thus, FABP1 could be a novel biomarker of psoriasis, perhaps its comorbidities, but not of its severity. In the absence of similar data for comparison, we can cite studies which showed crucial role of FABP1 in other systemic diseases, especially in renal and liver disorders [7, 20, 21, 34, 38]. Cakir et al. noted significantly higher serum liver-type FABP levels in patients with acute hepatitis, hepatic encephalopathy and stable cirrhosis and proved that FABP1 is strongly related to liver damage [7]. Karvellas et al. showed increased level of liver-type FABP in patients with Acetaminophen (APAP)-induced Acute Liver Failure (ALF) and its impact on poorer survival suggesting it may have good potential to discriminate survivors from non-survivors [20]. Further, Shi et al. demonstrated significant positive correlations between FABP1 and lipid profile, BMI, glucose homeostatic parameters in all the study subjects [34]. Despite the expected similar dependencies, we have not shown any significant relationship between liver-type FABP and metabolic parameters like lipids levels nor fasting glucose level or BMI, beside a positive correlation with the one after treatment. Thus, on the one hand, it undermines the predictive role of liver fatty acid-binding protein in evaluating the risk of CMDs in psoriatics. On the other hand, despite the lack of statistical significance between serum FABP1 concentration and metabolic indices or BMI, the protein level was somehow dependent on adiposity. Especially obese patients with psoriasis had the highest levels of liver-type FABP and markedly increased comparing with overweight or normal-weight ones and the healthy controls, also after treatment. In line are other valuable studies which have demonstrated on FABP1 gene-ablated mice that the protein could protect against age-, diet- or Western-diet induced obesity and hepatic steatosis [2, 28]. Our results are consistent with the study of Shi et al. who reported increased FABP1 in healthy obese persons compared with normal-weight ones pointing its role in obesity and insulin-resistance-related metabolic diseases [34]. The authors stressed that such paradoxically elevated liver fatty acid-binding protein level in obese persons could be a compensatory mechanism in response to the systemic inflammation or be due to resistance to FABP1 induced by obesity [34]. The compensatory FABP1 up-regulation should be considered also in psoriasis, however it may have a more complex ground, looking at multidirectional relationships with obesity or other various modified effectors influencing different inflammatory or immunological stimuli.

Worth mentioning here is polymorphism in the human FABP1 gene which may have impact on divergent outcomes. A number of studies noted conflicting correlations of T94A variant expression with BMI and dyslipidemias, which were both positive and negative, or unchanged [13, 30, 39]. Thus, we can assume that also phenotypic changes in the FABP family may influence obesity indices and FABPs biologic functions and their role in the development of certain diseases, including psoriasis.

Of the liver enzymes, only a positive correlation with ALT level after therapy was noted, reflecting rather doubtful role of FABP1 in predicting liver damage in psoriasis patients. At this point we can quote briefly strong positive correlations between serum and urine FABP1 and AST (aspartate transaminase) or ALT noted by Cakir et al. [7]. However the study group included patients with acute hepatitis, thus initially with hepatic dysfunction and markedly elevated liver enzymes activities [7]. Certainly, further in-depth research is required.

As for demographic and clinical measurements, our results did not shown any associations between FABP1 and disease duration, weight, height or gender of the study group, however it marginally correlated with age. The results from the literature are divergent and not related with psoriasis. No statistical significance in terms of gender was reported in patients with other systemic diseases [7, 34]. Cakir et al. did not report any statistically significant difference in terms of age in patients with acute hepatitis and healthy controls [7]. On the other hand, Shi et al. noted a meaningful negative relation of FABP1 with age in all the study group composed of obese and non-obese subjects [34].

Interestingly, evaluated FABP negatively correlated with certain blood morphotic elements such as RBC, HCT and HGB, also after therapy. To date, these are the first reported links between serum liver fatty acid-binding protein and anemia indices among patients with psoriasis. No similar data are available, however there are papers which have demonstrated this relation, but between urinary-FABP1 level and anemia in diabetic and non-diabetic subjects [19, 36]. Our notably correlations have some limitations and additional assessment of indices of iron status in psoriatics in relation with fatty acids metabolism would be valuable. The more, psoriasis has been linked with iron deficiency and anemia [31].

Further, our data demonstrate that liver fatty acid-binding protein significantly correlated with CRP along with statistically increased levels of the protein in moderate and severe psoriatics we might assume that FABP1 could be a potential mediator of inflammation, so profusely present in psoriasis. Kim et al. investigated the biological functions of FABP1 in patients with end-stage renal disease, though with other methods, and they suggested its role in systemic inflammation [21].

To date, there are no published investigations on the systemic antipsoriatic treatment on serum liver fatty-acid binding proteins. Both methotrexate and acitretin with a proven efficacy and extensive experience have been used against psoriasis over years. We aimed to assess the relations with FABP1 because of multidirectional effects of both drugs on psoriatics. Firstly, MTX through inhibiting chronic inflammation acts anti-atherosclerotic and lowers the risk of CVD and MI. On the other hand, methotrexate has also pro-atheromatous effect due increasing homocysteine level which is one of the oxidative stress markers enhanced in psoriasis [4, 8]. When planning the present study, we also took into account the well-known risk of hepatotoxicity of MTX, hence the additional curiosity to study its effect on liver-type FABP in our population.

Twelve-week of total therapy significantly decreased liver fatty acid-binding protein level, pointing to its relevance in monitoring treatment efficacy. FABP1 remained statistically higher than of the healthy controls. This outcome shows that despite the potential involvement of FABP1 in modulation of the risk of CMDs, the interplay along with the therapy is still insufficient. Although after separation of the study group no significant dependence with each drug was found, serum FABP1 level decreased over twice after acitretin while almost no change was observed after MTX. These observations arouse curiosity, when considering methotrexate’s impact on liver function, initially we assumed a significant effect of this drug on the concentration of liver-type FABP. Notable is that FABP1 level in psoriatics with obesity remained significantly higher after treatment pointing to close relation with adiposity. Perhaps, it would be worth to include dietary restrictions to reduce body weight, which together with the interplay between FABP1 and systemic therapy would reduce the risk of CMDs in psoriatics. Ligand binding properties of FABPs family along with enhancing expression of FABP1 by hypolipidemic drugs or antidiabetic thiazolidinedione (TZD) have been documented [7, 33, 40]. Considering that elevated serum FABP1 might be a marker of several CMDs and psoriasis, as we already have proved, lowering its levels would be desirable in psoriasis. Systemic therapy is effective, but not enough sufficient. Presumably connecting antipsoriatic treatment additionally with other lowering FABP1 medications could be useful. Worth mentioning here are effective different therapeutic strategies reducing adipocyte fatty acid-binding protein as a potential method against heart failure or other metabolic-related diseases [22, 32]. The literature data has presented efficacy of synthetic adipocyte-type FABP inhibitors, neutralizing FABP4 antibodies such as BMS309403, as well as other commonly used drugs such as angiotensin II receptor blockers or levofloxacin [15, 37]. Although further research on effectiveness and safety of such therapeutics is needed, these data highlights their potential use against CMDs development in psoriasis. Hypothesizing, also other methods as perhaps genetic modifications of FABP1 gene could improve the overall disruptions in psoriasis.

There are some limitations of our study. The study group is relatively small, however the evaluation was performed at two time points. This also enabled a deeper analysis of each drug’s influence on severity or BMI sub-groups. Mentioned earlier, the lack of certain laboratory indices limits the interpretation of some cause-effect relations. Presumably different behavioral, genetic or dietary factors could influence the results obtained in our population. We plan to conduct extended research of larger study group, different methods of treatment, longer follow-up period and further isoforms of FABPs.

## Conclusions

In summary, the present study showed for the first time that liver fatty acid-binding protein might serve as novel biomarker of psoriasis and a potential link with cardiometabolic comorbidities. Although we did not prove a clear relationship between metabolic parameters and FABP1, it should be somehow related with adiposity in psoriasis. Further, liver fatty acid-binding protein might be an indicator of systemic inflammation in psoriatics. Evaluated FABP is not useful in assessing the psoriasis activity, however might be important in severe type of the disease. Notable correlation with morphotic blood elements emphasizes the unknown relationship of FABP1 with anemia or other hematological disturbances in psoriasis. Systemic treatment significantly decreases serum liver-type FABP level reflecting the protein might serve as a predictor of clinical response to antipsoriatic therapy and minimize the risk of comorbidities development in psoriasis.

Taking into account the limitations of this work, the lack of data from the literature to reference, the results obtained can not be treated as enough conclusive and final. However, our outcomes are undoubtedly valuable by proving markedly important association of liver fatty acid-binding protein with psoriasis and its treatment, possible links with inflammation and comorbidities. They also rise the need to further clarify the precise role of fatty acid-binding proteins in psoriasis and their potential to predict or even treat immunometabolic diseases.
